# Investigating miR-9 as a mediator in laryngeal cancer health disparities

**DOI:** 10.3389/fonc.2023.1096882

**Published:** 2023-04-04

**Authors:** Christina Gobin, Samuel Inkabi, Chayil C. Lattimore, Tongjun Gu, James N. Menefee, Mayrangela Rodriguez, Heather Kates, Christopher Fields, Tengfei Bian, Natalie Silver, Chengguo Xing, Clayton Yates, Rolf Renne, Mingyi Xie, Kristianna M. Fredenburg

**Affiliations:** ^1^ Department of Pathology, Immunology, and Laboratory Medicine, University of Florida, Gainesville, FL, United States; ^2^ College of Graduate Health Studies, A.T. Still University, Kirksville, MO, United States; ^3^ Interdisciplinary Center for Biotechnology Research Bioinformatics Core Facility, University of Florida, Gainesville, FL, United States; ^4^ Department of Biochemistry and Molecular Biology, Baylor College of Medicine, Houston, TX, United States; ^5^ Department of Medicinal Chemistry, University of Florida, Gainesville, FL, United States; ^6^ Head and Neck Institute/Lerner Research Institute, Cleveland Clinic, Cleveland, OH, United States; ^7^ Department of Pathology, Johns Hopkins School of Medicine, Baltimore, MD, United States; ^8^ Sidney Kimmel Comprehensive Cancer Center, Johns Hopkins University School of Medicine, Baltimore, MD, United States; ^9^ Department of Urology, Johns Hopkins University School of Medicine, Baltimore, MD, United States; ^10^ Department of Molecular Genetics and Microbiology, University of Florida, Gainesville, FL, United States; ^11^ Department of Biochemistry and Molecular Biology, University of Florida, Gainesville, FL, United States

**Keywords:** cancer health disparities, laryngeal squamous cell carcinoma, miR-9, head and neck cancer, ABCC1, MAP1B

## Abstract

**Background:**

For several decades, Black patients have carried a higher burden of laryngeal cancer among all races. Even when accounting for sociodemographics, a disparity remains. Differentially expressed microRNAs have been linked to racially disparate clinical outcomes in breast and prostate cancers, yet an association in laryngeal cancer has not been addressed. In this study, we present our computational analysis of differentially expressed miRNAs in Black compared with White laryngeal cancer and further validate microRNA-9-5p (miR-9-5p) as a potential mediator of cancer phenotype and chemoresistance.

**Methods:**

Bioinformatic analysis of 111 (92 Whites, 19 Black) laryngeal squamous cell carcinoma (LSCC) specimens from the TCGA revealed miRNAs were significantly differentially expressed in Black compared with White LSCC. We focused on miR-9-5 p which had a significant 4-fold lower expression in Black compared with White LSCC (p<0.05). After transient transfection with either miR-9 mimic or inhibitor in cell lines derived from Black (UM-SCC-12) or White LSCC patients (UM-SCC-10A), cellular migration and cell proliferation was assessed. Alterations in cisplatin sensitivity was evaluated in transient transfected cells *via* IC50 analysis. qPCR was performed on transfected cells to evaluate miR-9 targets and chemoresistance predictors, ABCC1 and MAP1B.

**Results:**

Northern blot analysis revealed mature miR-9-5p was inherently lower in cell line UM-SCC-12 compared with UM-SCC-10A. UM -SCC-12 had baseline increase in cellular migration (p < 0.01), proliferation (p < 0.0001) and chemosensitivity (p < 0.01) compared to UM-SCC-10A. Increasing miR-9 in UM-SCC-12 cells resulted in decreased cellular migration (p < 0.05), decreased proliferation (p < 0.0001) and increased sensitivity to cisplatin (p < 0.001). Reducing miR-9 in UM-SCC-10A cells resulted in increased cellular migration (p < 0.05), increased proliferation (p < 0.05) and decreased sensitivity to cisplatin (p < 0.01). A significant inverse relationship in ABCC1 and MAP1B gene expression was observed when miR-9 levels were transiently elevated or reduced in either UM-SCC-12 or UM-SCC-10A cell lines, respectively, suggesting modulation by miR-9.

**Conclusion:**

Collectively, these studies introduce differential miRNA expression in LSCC cancer health disparities and propose a role for low miR-9-5p as a mediator in LSCC tumorigenesis and chemoresistance.

## Introduction

Black Americans carry a higher burden of head and neck squamous cell carcinoma (HNSCC) compared with other races (1–3). The underlying cause is multifactorial. In part, the disparities may be explained by differences in social determinants of health, including socioeconomic status, access to care, education and literacy (4–6). Clinically, Black patients present with HNSCC at younger age, have higher stage cancers, are more likely to present with advanced cancers (T-stage, N-stage) and while mortality rates have for HNC have decreased, a higher mortality rate remains for Black Americans relative to other races (7, 8).

The most commonly involved anatomic sites include the oral cavity, oropharynx, and larynx. Among the three, laryngeal squamous cell carcinoma (LSCC) harbors the lowest 5-year survival rate; moreover, treatment-related comorbidities and loss of quality of life also have resulted in some of the highest rates of suicide (1, 9). Black Americans have maintained a higher incidence of LSCC for over several decades, presenting with a greater likelihood of advanced stage disease and increased mortality (10–12). It is no surprise that socioeconomic and environmental factors play a role in these disparate clinical outcomes, however, after controlling for these factors, a disparity persists (13–15). There are few studies that have considered biology as a contributing factor to LSCC clinical disparate outcomes (16). Moreover, there are none that have explored the role of noncoding RNAs.

microRNAs (miRNAs) are small noncoding RNAs that are prominent players in many physiologic and pathologic processes including cell differentiation, proliferation, and survival (17). Understandably, deregulation of miRNAs can have a profound impact on cellular regulation and gene expression. They have been found to contribute to tumor development and metastasis in many cancers including LSCC (17–19). Differential expression of miRNAs has been found to be a feature of cancers where Blacks carry an unequal burden and have poorer outcomes (20–25). These studies have demonstrated that these biologic mediators and their targets vary by race and ethnicity (26). In addition, these reports suggest that differences in miRNA expression may explain disparate clinical outcomes in Black patients and may be exploited for their prognostic and predictive value (27, 28).

Here, we present our bioinformatic analysis of miRNAs in LSCC using the Cancer Genome Atlas where we use it to explore differential expression of miRNAs in Black compared with White patients. From this analysis, we turned our attention to investigating the role of miR-9-5p. Using two race-specific cell lines, we explore potential role of mir-9 in modulating a malignant phenotype cell and influencing chemoresistance. Finally, we explore the gene expression of two known targets of miR-9. To our knowledge, this is the first study of its kind in head and neck cancer, specifically LSCC. Overall, evaluating differential miRNA expression in the context of LSCC cancer health disparities and subsequently investigating their role as potential mediators of disease may provide opportunities to clinically predict treatment response and survival.

## Methods

### Bioinformatic analysis

Alignment files (bam files) for 92 White and 19 Black LSCC patients were downloaded from The Cancer Genome Atlas Head-Neck Squamous Cell Carcinoma (TCGA-HNSC). All bam files were converted to fastq files using bedtools (29). The raw reads from fastq files were preprocessed using mapper.pl from miRDeep2 (30). Quality control was performed by removing reads with alphabets other than a, c, g, t, u, n, A, C, G, T, U, N and reads less than 15 nucleotides long from downstream analysis. The remaining reads were aligned to human miRNA precursors downloaded from miRBase release 21 (31) using quantifier.pl from miRDeep2. Alignment between precursor and mature miRNA was performed to generate the final miRNA counts. In doing this, mature miRNA sequences were first downloaded from miRbase and aligned to their miRNA precursors. Then, the alignment between mature miRNAs and the reads were compared and the number of reads falling within 2nt upstream and 5nt downstream of the corresponding miRNA was taken as the read counts for that miRNA.

Differential expression analysis was performed to compare miRNA expression between Black and White tumor samples. Counts were normalized using Relative Log Expression (RLE) implemented from edgeR (32). A negative binomial generalized log-linear model implemented in edgeR was used for differential analysis. EdgeR differential expression analysis was performed using the White tumor group as the reference by default; the direction of the fold-change was reversed *post-hoc* to consider changes in Black tumor samples relative to White tumor samples. Significantly differentially expressed miRNAs identified at a fold change >1.5 and a p value < 0.05.

Sex was not considered as a biological variable due to the limited sample size of females within the TCGA dataset used for analysis. Randomization of the TCGA cohort was irrelevant because the study was specifically designed to explore differential miRNA expression by race. Blinding was also deemed irrelevant to the study design. Power analysis was not conducted for the RNA-Seq TCGA patient data because our exploratory data analysis was constrained by the limited data points available within the database following race stratification (92 White versus 19 Black).

### Laryngeal cancer cell lines

Human laryngeal squamous cell carcinoma cell lines, UM-SCC-12 (Black patient derived; RRID: CVCL_7717) and UM-SCC-10A (White patient derived; RRID: CVCL_7713) were authenticated *via* short tandem repeat typing and further genetically characterized (33, 34) prior to purchase from the University of Michigan Head and Neck cell line repository. Both cell lines were age, sex, grade and stage matched (see **Supplementary Table 1**). Cells were cultured in a T 75cm^2^ flask containing Dulbecco Modification of Eagle’s Medium 1X (DMEM, 10-013-CV, Corning) supplemented with 10% heat inactivated fetal bovine serum (FBS, 35-011-CV, Corning), and 2% Penicillin/Streptomycin (PENSTREP, 15-140-122, Gibco) within a humidified incubator containing 5% CO_2_ at 37°C. Cells were utilized in the following assays upon reaching 80% confluence.

### miRNA Northern blot

#### Preparation of IR labeled probes

IrNorthern probe sequences for U6, miR-191-5p, miR-9-5p, miR-16 and let7a (see **Supplementary Table 2**). Probes wereconjugated with DBCO-IR dye and then were purified by AMPure XP beads as previously described (35, 36).

#### Northern blot analyses

Northern blot analyses were performed using near infrared dye-labeled probes as previously described (35, 36). Briefly, 15 µg of total RNA from either UM-SCC-10A or UM-SCC-12 was separated using 15% Urea-PAGE and contents were subsequently transferred to Hybond N+ membrane (GE) using LifeTech transfer module at 0.2 Amp for one hour. The membrane was crosslinked twice using 254nm UV crosslinker at 120 mJ/cm^2^. The membrane was then placed in a hybridization oven and incubated with 10ml ExpressHyb hybridization solution (Takara) in a hybridization tube for 30 minutes at 30°C. IR-dye labeled probes and the membrane were then hybridized overnight at 30°C. After overnight hybridization, the membrane was washed twice, with 2x SSC buffer containing 0.1% SDS and 1x SSC buffer containing 0.1% SDS, respectively. For both washes, membrane was shaken at 110 rpm for 10 minutes at room temperature. Following washes, membrane was scanned on Amershan Typhoon scanner (GE health) to detect emission at 600 nm and 800 nm.

### Transient transfection

In a 12-well format, UM-SCC-12 or UM-SCC-10A cells were reverse transfected with 50nM of either miR-9 mimic or inhibitor, respectively (see **Supplementary Table 3** for oligo sequences and product information). Transfection efficiency was enhanced through the use of Lipofectamine RNAiMAX (13778075, Thermo Fisher) and Opti-MEM I Reduced Serum Medium (31985062, Thermo Fisher) per manufacturer’s instructions. Cell growth was optimized to ensure 60-80% confluency by assay endpoint. Length of transfection was dependent upon validation assay performed. All assays were performed in triplicate.

### Scratch wound assay

The scratch wound assay was used to assess cell migration. 24 hours post transfection, the confluent cell monolayer was disrupted using a 1000µL pipet tip. Images were captured at 0h, 24h, 48h, and 72h post cell monolayer disruption using the EVOS FL Cell Imaging System (ThermoFisher). The wound healing size tool plugin for ImageJ (RRID: SCR_003070) was used to quantify wound healing at each timepoint.

### Cisplatin IC50 assay

Baseline IC50 for cisplatin in UM-SCC-12 and UM-SCC-10A was determined with serial dilutions of Cisplatin (1134357, Millipore Sigma) at concentrations of 200µM, 66.67µM, 22.22µM, 7.41µM, 2.47µM, and 0µM. 48 hours post cisplatin treatment, cisplatin and spent media were aspirated from the wells and a 1:7 dilution of the Cell Titer Blue reagent (G8080, Promega) was applied to the cells in the wells. Baseline fluorescence (560/590 nm) was assessed using the BioTek SYNERGY H1 Multi-Mode Microplate Reader. The gain was adjusted such that all baseline values were similar ~2000nm. The plates were incubated in 5% CO_2_ at 37°C with subsequent plate readings taken in 30 min intervals for 4 hours. IC50 to cisplatin was calculated in GraphPad Prism Version 9.40 (RRID: SCR_002798) using percentage of cell viability values. Percentage of cell viability was calculated as follows:


$$\begin{array}{l} \left(\mathrm{Average final fluorescence values}\;-\;\mathrm{average baseline fluorescence values}\right)\;=\text{x}\\ \left(\text{x}\;-\;\mathrm{average fluorescence values of media only wells}\right)\;=\text{y}\\ \left(\text{y}÷\;\mathrm{average control fluorescence values of 0µM dose}\right)\;{\;}^{*}\;100\;=\;\%\;\mathrm{cell viability} \end{array}$$

### Cisplatin IC50 LSCC cell lines after transient transfection

24 hours post transfection with a specific oligo and appropriate control, cells were washed with 1mL of 1x dPBS (1x dPBS, 21-031-CV, Corning) per well. Cells were harvested with 1x TrypLE (12604-013, Gibco), counted and replated as five replicates per condition in a 96-well plate. The next day cells were treated as described above at 48 hours cisplatin treatment.

### Cell Proliferation in transient transfected cells

Average fluorescence values generated at no treatment dose (0µM of cisplatin) in transfected cells corresponded with baseline cell proliferation. As such, final average fluorescence values at the 0µM dose were normalized against their baseline average fluorescence values to calculate relative fluorescence which represented cell proliferation in transfected cells.

### Reverse transcriptase-PCR

24 hours post transfection, cells were washed with 1mL 1x dPBS per well and collected in cold TRIzol Reagent (15596018, Thermo Fisher). RNA was isolated per manufacturer’s instruction. RT-PCR was performed with the Eppendorf Mastercycler gradient to synthesize cDNA from 50ng/µL of RNA and random primers from the high-capacity cDNA reverse transcription kit (4368814, Applied Biosystems).

### qPCR

A 1:50 dilution of cDNA was combined with EXPRESS SYBR GreenER qPCR Supermix reagents (11784200, Thermofisher Scientific) and 2µM primer pairs of GAPDH, ABCC1 or MAP1B (see **Supplementary Table 4** for primer product information). qPCR experiments were run in triplicate with three biological replicates per condition using Applied Biosystems StepOne Plus Real time PCR system. CT values were normalized against corresponding GAPDH CT values using the 2^-ΔCT^ method and log transformed. A Methods schematic depicts the above described miRNA validation, chemosensitivity, and qPCR assays (**Supplementary Figure 1**). 

### Statistical analysis

All data were analyzed using GraphPad Prism (Version 9.40) software, setting the alpha level at 0.05 for all statistical analyses used. All experiments were completed using biological and technical replicates in triplicate. Two-way repeated measures ANOVAs were conducted to assess group differences across time points or drug doses between cell lines or across transfection conditions. A significant phenotypic change x cell line interaction was followed up with Šídák’s multiple comparison tests. Paired and unpaired t-tests were performed to assess group differences on single dependent measures when appropriate.

Biological sex was not considered due to the limited sample size of females per racial group in the LSCC TCGA dataset. Randomization and blinding of the TCGA cohort were irrelevant because the study was specifically designed to explore differential miRNA expression by race. Power analysis was not performed as this our study is exploratory and data points are limited.

## Results

### miRNAs are differentially expressed in Black compared with White laryngeal cancer

**Figure 1:**
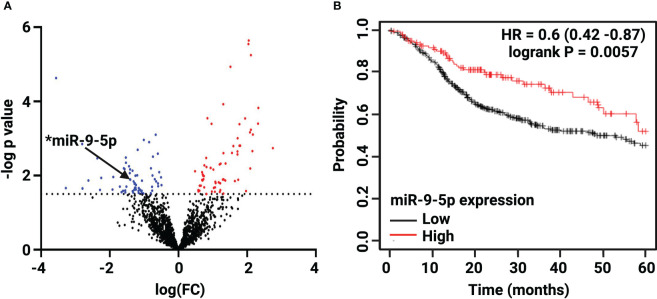
Differential expression of miR-9-5p in Black compared with White LSCC by TCGA analysis. **(A)** The Volcano plot depicts 132 differentially expressed miRNAs: 64 higher (red) and 68 lower (blue) in Black LSCC patients. The black arrow highlights low levels of miR-9-5p identified in Black LSCC patients (*p < 0.05). **(B)** Low miR-9-5p predicts poor overall survival in HNSCC as shown by Kaplan Meier survival curve (HR=0.6, logrank p=0.0057).

Bioinformatic analysis of the 92 White and 19 Black LSCC patients abstracted from the TCGA revealed 132 out of 1902 miRNAs were significantly differentially expressed (FC >1.5, *p* < 0.05) (**Supplementary Tables 5**, **6**) in Black compared with White LSCC. The volcano plot depicts (**Figure 1A**) a slightly greater number of miRNAs that are significantly lower (68 miRNAs) than higher (64 miRNAs) in Black compared with White LSCC patients. **Table 1** shows the top 30 miRNAs that are lower and higher in Black compared with White LSCC patients.

**Table 1:** Top 30 higher and lower significantly differentially expressed miRNAs in Black compared with White Laryngeal squamous cell carcinoma (LSCC).

miRNA	P-Value	log(FC)	miRNA	P-Value	log(FC)
miR-519a-5p	2.26E-06	2.045041	miR-3180-5p	2.34E-05	-3.56514
miR-518e-5p	2.81E-06	2.038477	miR-21-3p	0.000794	-0.66342
miR-4482-3p	5.61E-06	2.10879	miR-149-5p	0.001089	-1.01261
miR-451a	1.16E-05	1.515035	miR-141-5p	0.001254	-0.85338
miR-144-5p	0.000119	1.303104	miR-876-3p	0.001402	-2.80519
miR-1283-3p	0.000149	2.328334	miR-149-3p	0.002006	-1.18755
miR-363-3p	0.000283	0.841252	miR-30b-3p	0.002554	-0.60359
miR-520a-5p	0.000285	1.941621	miR-200c-5p	0.003081	-0.74309
miR-522-3p	0.000394	2.318118	miR-3155a	0.003242	-1.53699
miR-20b-5p	0.0004	0.963183	miR-934	0.003395	-2.36473
miR-4482-5p	0.000412	1.774413	miR-3691-3p	0.004493	-1.52952
miR-518a-5p	0.000576	2.107811	miR-6087	0.005605	-1.44064
miR-517-5p	0.00078	2.168416	miR-27a-5p	0.006322	-0.80296
miR-526b-5p	0.000984	1.724201	miR-762	0.006354	-1.59308
miR-520a-3p	0.001552	1.773994	miR-6742-3p	0.006649	-1.57277
miR-1323	0.001559	1.799962	miR-4524a-3p	0.006825	-1.41181
miR-518f-5p	0.00159	1.598958	miR-4270	0.007384	-1.34068
miR-514b-5p	0.001809	2.749585	miR-3913-5p	0.00808	-0.56311
miR-521	0.002181	2.152277	miR-190a-3p	0.008571	-1.42109
miR-525-5p	0.002237	1.716953	miR-585-3p	0.009817	-1.20301
miR-518d-5p	0.002445	1.559388	miR-335-3p	0.009953	-0.75986
miR-372-3p	0.002463	1.310864	miR-219b-5p	0.010027	-1.25389
miR-516a-5p	0.002668	1.715929	miR-371b-5p	0.010853	-1.48457
miR-154-3p	0.003342	0.807869	miR-891a-5p	0.0109	-1.89919
miR-4732-3p	0.00472	1.369273	miR-128-1-5p	0.011135	-0.5085
miR-655-5p	0.004743	1.226723	miR-5683	0.011569	-2.25301
miR-1299	0.005	1.19916	miR-210-5p	0.012541	-0.69138
miR-548j-5p	0.005283	0.752899	*miR-9-5p	0.012751	-1.40489
miR-153-3p	0.005876	0.816662	miR-4289	0.013638	-2.62336
miR-486-5p	0.00618	0.935478	miR-7974	0.013759	-1.0649

We focused on miR-9-5p as it is one of the more abundant miRNAs that also has been characterized as a potential biomarker in head and neck cancer (37). In addition, a survival curve generated by Kmplotter (38) in **Figure 1B** shows low miR-9 levels correlate with poor overall survival in HNSCC (HR=0.6, logrank p=0.0057). As one of the top 30 lower expressed miRNAs, miR-9 was found to be 4-fold lower (log FC =-1.41) in Black compared with White LSCC patients at *p* = 0.013.

### Characterization of miR-9-5p in Black and White patient-derived LSCC cell lines by Northern blot

**Figure 2:**
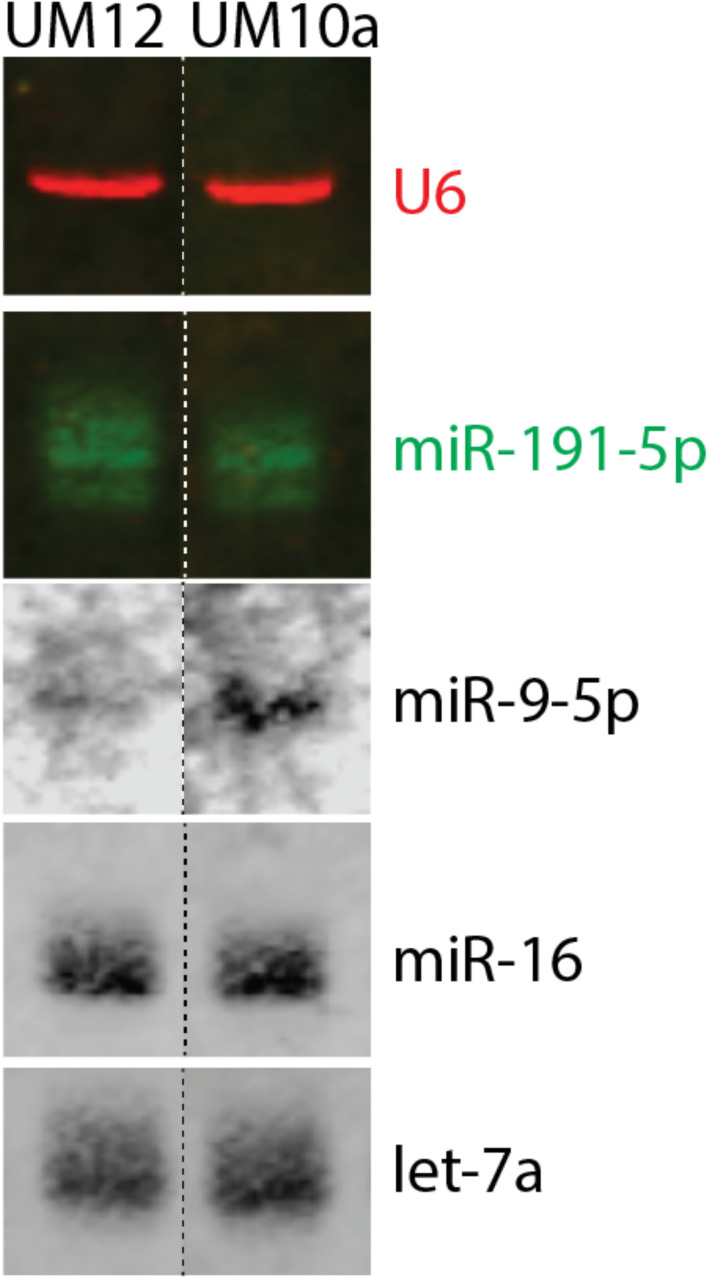
Characterization of miR-9-5p in race-specific LSCC cell lines. Northern blot analysis revealed that Black patient derived UM-SCC-12 cell line has barely detectable levels of mature miR-9-5p whereas the White-patient derived UM-SCC-10A cell line has a greater level of mature miR-9-5p. U6, miR-191-5p, miR-16, and Let 7A were used as internal loading controls.

To explore the influence of miR-9-5p in the context of race, we sought to identify cell lines derived from patients with similar clinicopathologic characteristics, differing only by self-reported race and miR-9-5p levels (**Supplementary Table 1**). We discovered two cell lines that fit those parameters, UM-SCC-12 (derived from a Black male patient) and UM-SCC-10A (derived from a White male patient) cell lines that were established at the University of Michigan. To assess the expression of mature miR-9-5p in the cell lines, we performed Northern Blot analysis. miR-9-5p was weakly detectable in the UM-SCC-12 cell line. Strong expression of miR-9-5p was detected in the UM-SCC-10A cell line (**Figure 2**). Endogenous U6, miR-191-5p, miR-16, and Let 7A served as internal loading controls.

### Characterization of UM-SCC-12 and UM-SCC-10A cellular phenotype and cisplatin chemosensitivity

To properly interpret our miR-9 validation results, we were required to first determine the intrinsic cellular behavior of the cell lines. We characterized baseline differences in cell migration, cell proliferation and chemosensitivity between our two cell lines.

**Figure 3:**
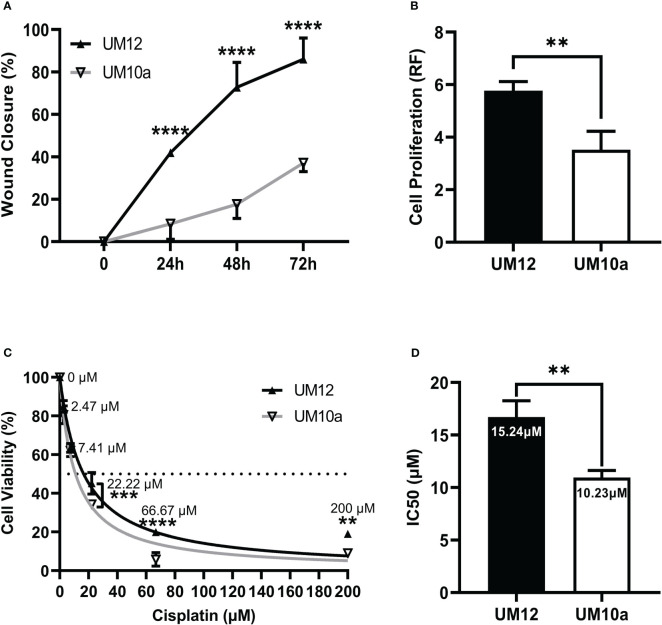
Characterization of UM-SCC-12 and UM-SCC-10A cellular phenotype and cisplatin chemosensitivity. UM-SCC-12 cells had significantly greater baseline **(A)** cell migration across 24h, 48h, and 72h time points **(B)** and cell proliferation, **(C)** decreased cell killing by cisplatin at 22.22 μM, 66.67μM and 200μM, and thus a **(D)** higher cisplatin IC50 when compared to UM-SCC-10A. **p < 0.01, ***p < 0.001, ****p < 0.0001.

Cell migration was assessed *via* the scratch wound assay and operationalized as percentage of wound closure 24h, 48h, and 72h post monolayer disruption. A two-way repeated measures ANOVA was conducted to assess differences in percentage of wound closure across each time point between the two cell lines. UM-SCC-12 cell line had a significantly greater cellular migration compared with UM-SCC-10A cell line at 24h, 48h, and 72h post monolayer disruption (*p* < 0.0001) by Šídák’s multiple comparisons test (**Figure 3A**).

Cell proliferation was assessed in both cell lines *via* cell titer blue fluorescence assay at 72h. An unpaired t- test was conducted to assess differences in relative fluorescence between the cell lines. **Figure 3B** shows that UM-SCC-12 cell line had a greater rate of proliferation at 72 hours compared with UM-SCC-10A cell line (t (4) = 4.96, *p* < 0.001).

As cisplatin is the primary chemotherapeutic treatment for head and neck cancer, we assessed baseline cisplatin IC50 for both cell lines. Cell line viability was measured at 48h of cisplatin treatment. A two-way repeated measures ANOVA was then conducted to assess differences in cell viability across the cisplatin concentrations between the two cell lines. UM-SCC-12 cell line had decreased sensitivity to cisplatin compared with UM-SCC-10A cell line at the three highest doses of cisplatin treatment: 22.22µM (*p* < 0.001), 66.67µM (*p* < 0.0001), and 200µM (*p* < 0.01) (**Figure 3C**).

An unpaired t-test was conducted on IC50 values generated from three experimental runs between the cell lines. A nonlinear fit of the normalized percentage of cell viability responses relative to the non-transformed cisplatin concentrations was used to calculate the IC50. The calculated IC50 of cisplatin was significantly greater for the UM-SCC-12 cell line at 15.24 um compared with 10.23 um for UM-SCC-10A (t(4) = 5.86, *p* < 0.01, **Figure 3D**).

Collectively, UM-SCC-12 cells had significantly greater baseline cell migration, cell proliferation, and decreased cell killing by cisplatin, and thus a higher cisplatin IC50 when compared to UM-SCC-10A.

### Increasing miR-9 decreases cell migration, cell proliferation and increases chemosensitivity

**Figure 4:**
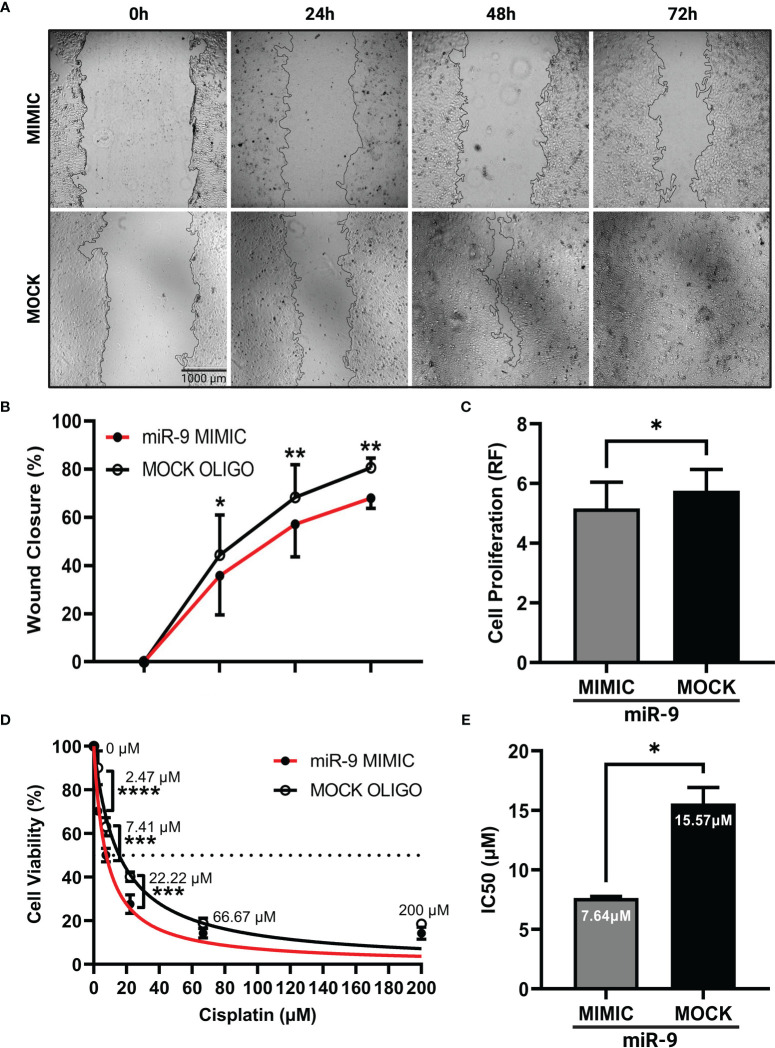
Increasing miR-9 decreases, cell migration, decreases cell proliferation, and increases chemosensitivity in UM-SCC-12 cells. **(A)** Representative images of cell migration of miR-9 mimic and mock oligo control transfected UM-SCC-12 cells across time points. Transient transfection of miR-9 mimic in UM-SCC-12 cells resulted in significantly decreased **(B)** cell migration at 24h, 48h, and 72h, **(C)** and cell proliferation, **(D)** increased sensitivity to cisplatin at 2.47 μM, 7.41μM and 22.22μM and **(E)** a lowering of its IC50 to cisplatin compared to mock oligo control. *p < 0.05, **p < 0.01, ***p < 0.001, ****p < 0.0001.

We next wanted to understand how increasing miR-9 levels in UM-SCC-12 cell line would alter its cellular phenotype. UM-SCC-12 cells were transiently transfected with a miR-9 mimic or mock oligo control. The scratch wound assay was performed. **Figure 4A** shows representative images of wound closure captured at 0h, 24h, 48h, and 72h post monolayer disruption for miR-9 mimic and mock transiently transfected cells. By multiple comparisons test, the mimic transfected UM-SCC-12 cells had a significantly lower percentage of wound closure compared to mock transfected control cells at 24h (*p* < 0.05), 48h (*p* < 0.01), and 72h post monolayer disruption (*p* < 0.01) (**Figure 4B**), demonstrating that elevated levels of miR-9 can decrease cellular migration in UM-SCC-12 cell line.

To assess cell proliferation in miR-9 transfected UM-SCC-12 cells, cell titer blue assay was performed on miR-9 transfected UM-SCC-12 cells. A paired samples t-test was conducted on relative fluorescence values across transfection conditions and revealed that the miR-9 transfected UM-SCC-12 cells exhibited lower relative fluorescence compared to mock oligo control, indicating a decrease in cell proliferation (t(2) = 4.65, *p* < 0.05) (**Figure 4C**).

We proceeded to test the effect of increasing miR-9 levels and chemosensitivity. miR-9 transfected UM-SCC-12 cells were treated with cisplatin and the IC50 was recorded. We noted an increase in cell killing/decreased cell viability in miR-9 transfected cells as compared to mock oligo control at three doses of cisplatin treatment: 2.47µM (*p* < 0.0001), 7.41µM (*p* < 0.001), and 22.22µM (*p* < 0.001) (**Figure 4D**). Furthermore, the calculated IC50 was lower (7.64 µM) compared to than mock oligo control (15.57 µM). Overall, these finding demonstrated that elevated levels of miR-9 can increase UM-SCC-12 sensitivity to cisplatin (**Figure 4E**).

### Reducing miR-9 increases cell migration, cell proliferation and decreases chemosensitivity

**Figure 5:**
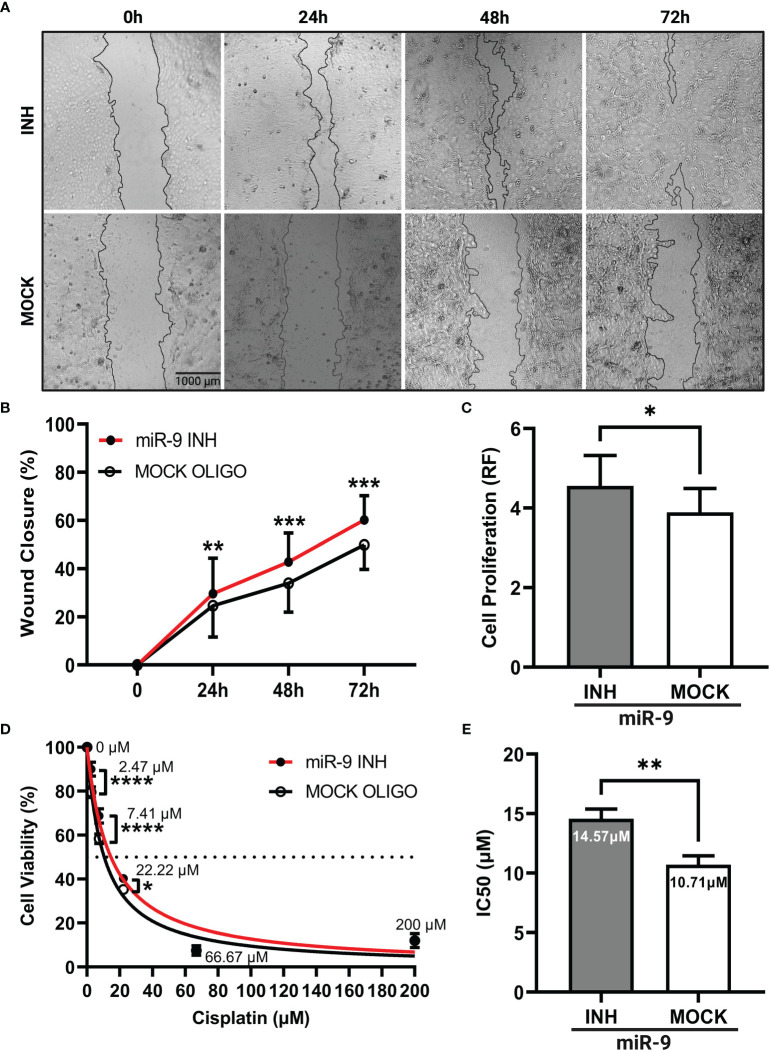
Reducing levels of miR-9 increases cell migration, increases cell proliferation, and decreases chemosensitivity in UM-SCC-10A cells. **(A)** Representative images of cell migration of miR-9 inhibitor or mock oligo control transfected UM-SCC-10A cells across time points. Transient transfection of miR-9 inhibitor in UM-SCC-10A cells resulted in significantly increased **(B)** cell migration at 24h, 48h, and 72h, **(C)** and cell proliferation, **(D)** decreased sensitivity to cisplatin at 2.47 μM, 7.41μM and 22.22μM and **(E)** an increase its IC50 to cisplatin compared to mock oligo control. *p < 0.05, **p < 0.01, ***p < 0.001, ****p < 0.0001.

Our next step was to determine if reducing miR-9 levels could produce an opposite phenotype as seen in our miR-9 transfected UM-SCC-12 cells. Here, we knockdown miR-9 levels by treating UM-SCC-10A cells with a miR-9 inhibitor. The scratch assay was performed. **Figure 5A** shows a panel of representative images of miR-9 inhibited UM-SCC-10A cells at 0h, 24h, 48h, and 72h compared with mock oligo control, showing lower miR-9 levels can significantly increase cell migration at each time point, 24h (*p* < 0.01), 48h (*p* < 0.001), and 72h post monolayer disruption (*p* < 0.001) (**Figure 5B**).

Cell proliferation was evaluated in miR-9 inhibited UM-SCC-10A transfected cells at 72 hours. Relative fluorescence values across transfection conditions was compared *via* paired t test. **Figure 5C** shows that cell proliferation was higher in miR-9 inhibited cells relative to the mock oligo control (t(2) = 4.94, *p* < 0.05).

We next compared chemosensitivity in miR-9 inhibited UM-SCC-10A transfected cells compared to mock oligo control. We found that lowering miR-9 levels decrease cell killing/decreased chemosensitivity at three doses of cisplatin treatment: 2.47µM (*p* < 0.0001), 7.41µM (*p* < 0.0001), and 22.22µM (*p* < 0.05) (**Figure 5D**). The IC50 values was higher (14.57 µM) compared to than mock oligo control (10.71 µM). These findings signify that lower miR-9 levels can decrease cellular sensitivity to cisplatin (t(2) = 28.79, *p* < 0.01) (**Figure 5E**).

### miR-9 modulates ABCC1 and MAP1B gene expression in LSCC cell lines

**Figure 6:**
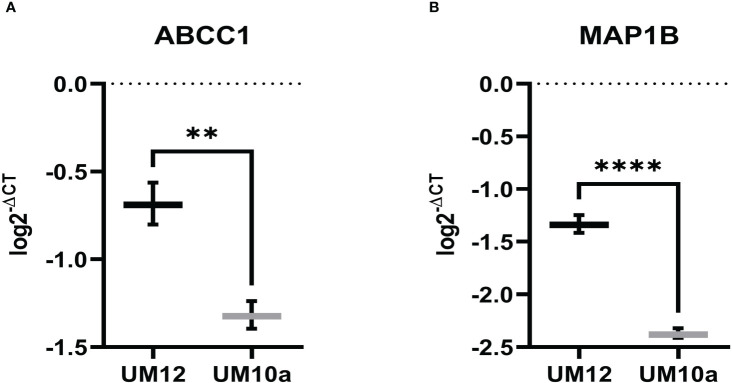
ABCC1 and MAP1B baseline gene expression in LSCC cell lines. **(A)** ABCC1 and **(B)** MAP1B gene expression is significantly higher in UM-SCC-12 cell line compare with UM-SCC-10A. **p < 0.01, ****p < 0.0001.

ABCC1 and MAP1B are reported gene targets of miR-9-5p that have been found to predict chemoresistance in cancer (39–42). Thus, as mediators of chemoresistance, we were interested in investigating whether these genes were regulated by miR-9 in LSCC. Initial studies involved determining baseline expression of ABCC1 and MAP1B in UM-SCC-12 and UM-SCC-10A by qPCR. Unpaired t-tests were conducted to assess differences in the log transformed 2^-ΔCT^ values between the two cell lines. UM-SCC-12 had significantly higher baseline gene expression of ABCC1 (t(4) = 7.69, *p* < 0.01) and MAP1B (t(4) = 18.69, *p* < 0.0001) compared with the UM-SCC-10A cell line (t(4) = 7.69, *p* < 0.01) (**Figures 6A, B**).

**Figure 7:**
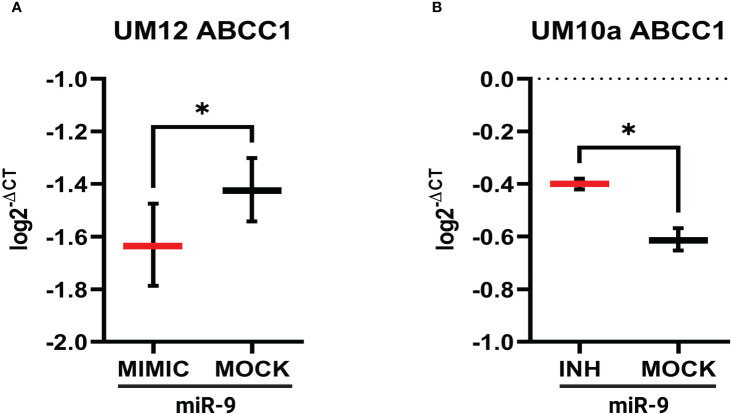
miR-9 modulates ABCC1 gene expression in LSCC cell lines. **(A)** Increasing miR-9 levels in UM-SCC-12 resulted in a significant decrease in ABCC1 gene expression compared with mock oligo control. **(B)** Decreasing miR-9 levels in UM-SCC-10A resulted in a significant increase in ABCC1 gene expression compared with mock oligo control. *p < 0.05.

Using miR-9 transfected UM-SCC-12 and miR-9 inhibited transfected UM-SCC-10A cells, we sought to determine if miR-9 regulated ABCC1 and MAP1B expression. 24 hours after transfection, gene expression for both ABCC1 and MAP1B was determined by qPCR. **Figure 7A** shows increased levels of miR-9 in UM-SCC-12 cells significantly decreased ABCC1 gene expression relative to the mock oligo control (t(2) = 4.63, *p* < 0.05). Conversely, reducing miR-9 levels in UM-SCC-10A significantly increased ABCC1 expression compared with mock oligo control ((t(2) = 7.42, *p* < 0.05) (**Figure 7B**).

**Figure 8:**
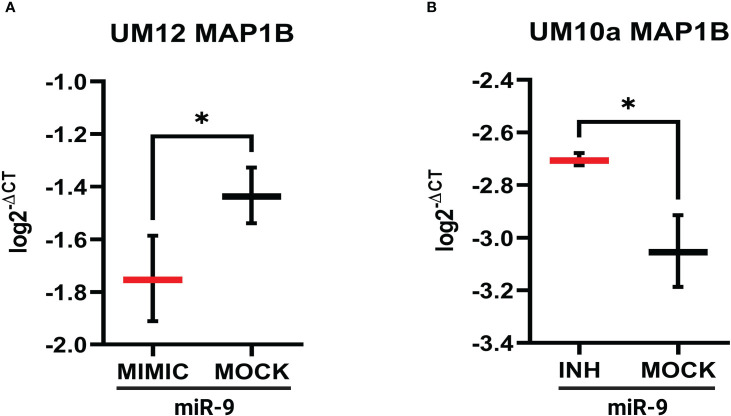
miR-9 modulates MAP1B gene expression in LSCC cell line. **(A)**. Increasing miR-9 levels in UM-SCC-12 resulted in a significant decrease in MAP1B gene expression compared with mock oligo control. **(B)** Decreasing miR-9 levels in UM-SCC-10A resulted in a significant increase in ABCC1 gene expression compared with mock oligo control. *p < 0.05.

Similar qPCR results were seen with MAP1B whereby increasing miR-9 in UM-SCC-12 cells reduced MAP1B gene expression relative to mock oligo control (t(2) = 6.19, *p* < 0.05, **Figure 8A**). **Figure 8B** shows that reducing miR-9 in UM-SCC-10A cells resulted in increased levels MAP1B relative to mock oligo control (t(2) = 5.22, *p* < 0.05). Taken together, these studies suggest that miR-9 regulates ABCC1 and MAP1B levels in LSCC cell lines.

## Discussion

Genomic studies investigating heritable somatic alterations associated with racially disparate clinical outcomes have been dedicated to breast, colon, and prostate cancers (43–45); however, genomic alterations in LSCC been largely unexplored where Black Americans are disproportionately affected, harboring the lowest 5-year survival rates among all races. Investigators have identified ancestral-related nucleotide signatures in key driver genes, in particular PIK3CA in Black and White LSCCs from the TCGA but characterization of noncoding RNAs, namely miRNAs, has not been explored (16). We employed computation analysis of LSCCs in the TCGA and uncovered a panel of miRNAs that were significantly different in Black LSCC compared with White. Through a series of validation studies, we investigated miR-9 in LSCC tumorigenesis and uncovered its ability to influence sensitivity to cisplatin and predict chemoresistance–employing race-specific cell lines.

Our study is the first to report differential miRNA expression in Black and White head and neck cancer, specifically laryngeal cancer. Our findings may facilitate the development of miRNA signatures for LSCC that are associated with race. miRNA signatures have been reported for multiple cancers and linked to differential signaling pathway activation in known oncogenic drivers that impact clinical outcomes. In multiple myeloma, for example, a signature of six upregulated miRs was associated with the WNT signaling pathway, whereas a signature of four downregulated miRs was associated with the MAPK pathway (46). Inamoto and colleagues determined from 84 urothelial cancer of the bladder (UCB) patients that there was specific signature of nine miRs associated with an aggressive phenotype compared with a nonaggressive phenotype. Furthermore, six of those miRs were associated with a high-risk UCB phenotype and poor outcomes, whereas a signature of 3 miRs was found to be protective (47). Multiple miRNA signatures have been identified and demonstrated as robust predictors for the diagnosis of LSCC (48); however, they have not been identified in the context of LSCC racial health disparities. Our laboratory is currently utilizing computational analysis to expand on our expression data by exploring differentially expressed miRNA mediated pathway differences in the context of race.

Of the differentially expressed miRNAs, we turned our attention to miR-9 for several reasons; we observed greater abundance of miR-9 in the TCGA samples in both Black and White compared with several of the other differentially miRNAs, making it ideal as a potential biomarker; its low expression has been found to predict poor overall survival (37); and its role appears critical to development and disease (49). Overall, our hope was to evaluate miR-9 as a potential mediator LSCC tumorigenesis while also considering it as a potential biomarker for cancer health disparities.

miR-9 is a key regulator of neuronal development; playing critical role in spatial and temporal regulation of neurogenesis (50). As a regulator of cancer development, miR-9 has been shown to promote a cancerous phenotype depending on its expression levels and tumor origin. As an example, elevated levels of miR-9 have been associated with development of cervical and brain cancers and downstream activation of CAM and JAK/STAT pathways, respectively (51, 52). Decreased levels of miR-9 have been linked to tumorigenesis of triple negative breast cancer and ovarian cancer were signaling pathways of NOTCH1 and NF-kB have been proposed to play a role in their tumorigenesis (53, 54). Overall, the versatile expression of this miRNA across cancers indicates that unique pathways are activated depending on its expression levels. Using the TCGA, we found that miR-9 was significantly lower in Black patient LSCC samples compared with White. We sought to validate the relevance of low miR-9 levels in laryngeal cancer in two patient-derived LSCC cells lines. Distinctly, we took into consideration reported racial background of the patient-derived cell line, matched the provided clinicopathologic data, and ensured by Northern analysis that expression differences corresponded to the TCGA findings. Our validation studies suggest that low miR-9 levels influence LSCC tumorigenesis *via* increases in cell proliferation and migration. Our findings are similar to studies in oral squamous cell carcinoma where miR-9 levels were found to be lower in tumor than normal paired tissues from Southeast Asian patients (55). The authors demonstrated that overexpression of miR-9 could decrease migration, proliferation, and arrest the cell cycle. Furthermore, low miR-9 has been proposed to mediate OSCC tumorigenesis through WNT and CDK4/6 signaling in OSCC (55, 56). The means of low miR-9 expression in HNSCC was addressed by Minor and colleagues who demonstrated, in both *in vivo* and vitro model systems, that hypermethylation could reduce miR-9 levels in oral and oropharyngeal cancers (57).

It is important to note others have found high levels of miR-9 in HNSCC *via* computational analysis of the TCGA (58). Our contrary findings may be attributed to our race-centered analysis. It has been reported that there is greater representation of White patients compared other races in the TCGA (59). As such, this allows the genomic profiles of White patients to overshadow the biologic differences extant in individuals from underrepresented groups. Our findings may highlight a described limitation of using one database to define tumor biology for an entire population (60).

On that note, we also understand that a limitation to our findings is the small sample size of Black LSCC patients within the TCGA. However, we believe, by investigating miR-9, we can begin to address the significance of differential miRNA expression in LSCC tumorigenesis with cancer health disparities in the forefront. Our findings are supported by studies in other racial disparate cancers where certain miRNAs have been characterized as potential mediators of cancer health disparities. Yates and colleagues were the first to identify differential expression of mir-26a in Black compared with White prostate cancer cell lines. miR-26a was found to be overexpressed 13-fold in Black tumors compared with White tumors (25). The authors suggested that higher expression was associated with more aggressive phenotype whereas low miR-26a expression was associated with better survival. Similar studies have been performed in colorectal cancer (CRC) and breast cancers. In CRC, miR-182 was found to be upregulated in Black American CRC and further associated with reduction of the miR-182 targets–FOXO1 and FOXO3A (61). As tumor suppressors, FOXO1 and FOXO3 were suggested to be downstream mediators of CRC cancer health disparities. Taken together, these studies introduce the concept that versatile expression of a miRNA is not only associated with tumor origin but may also be associated with race.

Cisplatin is the primary drug used to treat all head and neck cancers. Primary or acquired resistance to cisplatin is a major clinical challenge (62). Based on studies in oral cavity and hepatocellular cancers that showed low levels of miR-9 confer chemoresistance, we sought to further explore a potential role for miR-9 in LSCC disparate clinical outcomes by investigating its role in cisplatin chemosensitivity (63, 64). Indeed, we demonstrated in LSCC cell lines that lowering miR-9 levels can decrease cell killing in response to cisplatin and that increasing miR-9 can increase cell killing in response to cisplatin. These findings would suggest that low miR-9 may influence survival through its modulating cisplatin chemosensitivity, conferring a chemoresistant phenotype.

ABCC1 and MAP1B are recognized miR-9 gene targets that have a potential role of chemoresistance. ABCC1 is one of the most studied multidrug resistant proteins. Its overexpression has been associated with chemotherapeutic drug resistance, distant metastasis, and poor clinical outcomes (40). As such, it has a been touted as a putative marker or a multi-marker panel member to predict chemoresistance (65). MAP1B is a member of the family of proteins essential to stabilizing microtubules. Disrupting microtubule assembly is a common target for chemotherapeutic drugs. Overexpression of MAP1B has been found to correlate with adverse clinical outcomes and predict unfavorable prognostic factors in urothelial carcinoma and glioblastoma (66). Using our patient-derived cells, we showed that our Black patient derived cell line with low miR-9 levels had significantly higher levels of both ABCC1 and MAP1B compared with the White patient derived cell line with higher miR-9 levels. By modulating miR-9 levels we were able to significantly alter gene expression levels of ABCC1 and MAP1B, suggesting that these genes are targets of miR-9 in LSCC. Consequently, we may have identified potential miR-9 downstream mediators of LSCC cancer health disparities that may be exploited for future therapeutic intervention.

In summary, our study investigates miR-9 influence on the cancer cell phenotype and modulating chemoresistance in LSCC cell lines. We understand a primary limitation to our investigation is the small number of LSCC samples in the TCGA and we are currently validating these findings in an additional cohort of samples taking ancestry into account. Nevertheless, our work may open the door for new therapies for LSCC based on targets of differentially expressed miRNAs and the expression of specific downstream pathways. Elucidating the biologic mechanisms underlying LSCC clinical disparate outcomes may provide better avenues for treatment and reduce mortality for all patient suffering with this cancer.

## Data availability statement

Publicly available datasets were analyzed in this study. This data can be found here: https://portal.gdc.cancer.gov.

## Author contributions

CG: Technical work, writing of manuscript, review, editing, conceptualization, statistics. SI: Foundational technical work and methodology CL: Technical and computational work, writing, editing, TG: Computational work. JM and MR: Technical work. HK: Computational methodology. CF and TB: Technical instruction. NS: Conceptualization. CX: Methodology and technical support. CY: Foundational conceptualization. RR: Foundational work. MX: Technical work, methodology, and foundational work, writing and editing. KF: Primary conceptualization, technical work, writing of manuscript, editing, and review. All authors contributed to the article and approved the submitted version.
