# Supplementary material for: Investigating miR-9 as a mediator in laryngeal cancer health disparities
Source: Front Oncol. 2023 Apr 4;13:1096882. doi: 10.3389/fonc.2023.1096882 (PMC10112398; doi:10.3389/fonc.2023.1096882)
Supplement: Supplementary file 1 [file DataSheet_1.docx]

**Supplemental Table 1: Cell Line Characteristics**

|  | **UM-SCC-12** | **UM-SCC-10a** |
| --- | --- | --- |
| **Race** | Black | White |
| **Age** | 71 | 57 |
| **Sex** | Male | Male |
| **Grade** | MWD | MWD |
| **Stage** | III | III |
| **Site** | Larynx | Larynx |
| **Culture** | Explant | Explant |

**Supplemental Table 2: Northern blot probes**

| **Probe name** | **Sequence** | **Manufacturer** |
| --- | --- | --- |
| U6 | GCAGGGGCCATGCTAATCTTCTCTGTATCG/iAzideN/T | Integrated DNA Technologies |
| miR-191-5p | CAGCTGCTTTTGGGATTCCGTTG/iAzideN/C | Integrated DNA Technologies |
| miR-9-5p | TCATACAGCTAGATAACCAAAGA/iAzideN/A | Integrated DNA Technologies |
| miR-16 | CGC CAA TAT TTA CGT GCT GCT A/iAzideN/A | Integrated DNA Technologies |
| let7a | AAC TAT ACA ACC TAC TAC CTC A/iAzideN/A | Integrated DNA Technologies |

**Supplemental Table 3: Transfection oligonucleotides**

|  | **UM-SCC-12** | | **UM-SCC-10a** | |
| --- | --- | --- | --- | --- |
|  | **Mimic** | **Mock** | **Inhibitor** | **Mock** |
| **Product group** | HSA-miR-9-5P, miRCURY LNA miRNA mimic (5) | Negative Control 4, miRCURY LNA miRNA mimic (5) | HSA-miR-9-5P, miRCURY LNA miRNA Inhibitor (5) | Negative Control A, miRCURY LNA miRNA Inhibitor Control (5) |
| **miRNA strand** | UCUUUGGUUAUCUAGCUGUAUGA | GAUGGCAUUCGAUCAGUUCUA | TCATACAGCTAGATAACCAAAG | TAACACGTCTATACGCCCA |
| **Cat. no** | 339173 YM00471434-ADA, Qiagen | 339173 YM00479903-ADA, Qiagen | 339121 YI04100536-ADA, Qiagen | 339126 YI00199006-ADA, Qiagen |
| **Purpose** | Overexpress  miR-9 | Negative Control | Knockdown  miR-9 | Negative Control |

**Supplemental Table 4: Primer pair sequences for GAPDH, ABCC1 and MAP1B**

| **Primer name** | **Sequence** | **Manufacturer** |
| --- | --- | --- |
| GAPDH Forward | ACACCATGGGGAAGGTGAAG | Eurofins Genomics |
| GAPDH Reverse | GTGACCAGGCGCCCAATA | Eurofins Genomics |
| ABCC1 Forward | CCTGTTCTCGGAAACCATCC | Eurofins Genomics |
| ABCC1 Reverse | AAGGTGATCCTCGACAGGAA | Eurofins Genomics |
| MAP1B Forward | CAAGAATGCTGCCAATGCCT | Integrated DNA Technologies |
| MAP1B Reverse | AGCAGGGTCATTCCCACTCA | Integrated DNA Technologies |

|  | **miRNA** | **P-Value** | **log(FC)** |  | |  |  |
| --- | --- | --- | --- | --- | --- | --- | --- |
|  | miR-519a-5p | 2.26E-06 | 2.045041 |  | |  |  |
|  | miR-518e-5p | 2.81E-06 | 2.038477 |  | |  |  |
|  | miR-4482-3p | 5.61E-06 | 2.10879 |  | |  |  |
|  | miR-451a | 1.16E-05 | 1.515035 |  | |  |  |
|  | miR-144-5p | 0.000119 | 1.303104 |  | |  |  |
|  | miR-1283 | 0.000149 | 2.328334 |  | |  |  |
|  | miR-363-3p | 0.000283 | 0.841252 |  | |  |  |
|  | miR-520a-5p | 0.000285 | 1.941621 |  | |  |  |
|  | miR-522-3p | 0.000394 | 2.318118 |  | |  |  |
|  | miR-20b-5p | 0.0004 | 0.963183 |  | |  |  |
|  | miR-4482-5p | 0.000412 | 1.774413 |  | |  |  |
|  | miR-518a-5p | 0.000576 | 2.107811 |  | |  |  |
|  | miR-517-5p | 0.00078 | 2.168416 |  | |  |  |
|  | miR-526b-5p | 0.000984 | 1.724201 |  | |  |  |
|  | miR-520a-3p | 0.001552 | 1.773994 |  | |  |  |
|  | miR-1323 | 0.001559 | 1.799962 |  | |  |  |
|  | miR-518f-5p | 0.00159 | 1.598958 |  | |  |  |
|  | miR-514b-5p | 0.001809 | 2.749585 |  | |  |  |
|  | miR-521 | 0.002181 | 2.152277 |  | |  |  |
|  | miR-525-5p | 0.002237 | 1.716953 |  | |  |  |
|  | miR-518d-5p | 0.002445 | 1.559388 |  | |  |  |
|  | miR-372-3p | 0.002463 | 1.310864 |  | |  |  |
|  | miR-516a-5p | 0.002668 | 1.715929 |  | |  |  |
|  | miR-154-3p | 0.003342 | 0.807869 |  | |  |  |
|  | miR-4732-3p | 0.00472 | 1.369273 |  | |  |  |
|  | miR-655-5p | 0.004743 | 1.226723 |  | |  |  |
|  | miR-1299 | 0.005 | 1.19916 |  | |  |  |
|  | miR-548j-5p | 0.005283 | 0.752899 |  | |  |  |
|  | miR-153-3p | 0.005876 | 0.816662 |  | |  |  |
|  | miR-486-5p | 0.00618 | 0.935478 |  | |  |  |
|  | miR-524-5p | 0.006401 | 2.091455 |  | |  |  |
|  | miR-144-3p | 0.006899 | 1.015389 |  | |  |  |
|  | miR-140-5p | 0.007642 | 0.48325 |  | |  |  |
|  | miR-337-3p | 0.008108 | 0.686006 |  | |  |  |
|  | miR-5701 | 0.009318 | 0.705036 |  | |  |  |
|  | miR-628-3p | 0.01011 | 0.717951 |  | |  |  |
|  | miR-539-5p | 0.01033 | 0.741111 |  | |  |  |
|  | miR-150-3p | 0.012503 | 0.724457 |  | |  |  |
|  | miR-6850-5p | 0.012646 | 1.313535 |  | |  |  |
|  | miR-518c-3p | 0.012792 | 1.289899 |  | |  |  |
|  | miR-601 | 0.013019 | 1.741888 |  | |  |  |
|  | miR-485-3p | 0.013094 | 0.688809 |  | |  |  |
|  | miR-150-5p | 0.013493 | 0.702078 |  | |  |  |
|  | miR-517a-3p | 0.013585 | 1.363244 |  | |  |  |
|  | miR-523-3p | 0.014059 | 1.64429 |  | |  |  |
|  | miR-6826-3p | 0.014687 | 1.073618 |  | |  |  |
|  | miR-548a-5p | 0.015223 | 1.170355 |  | |  |  |
|  | miR-4521 | 0.015873 | 1.081683 |  | |  |  |
|  | miR-3939 | 0.016163 | 1.215659 |  | |  |  |
|  | miR-153-5p | 0.018714 | 0.651478 |  | |  |  |
|  | miR-500b-3p | 0.018718 | 0.616873 |  | |  |  |
|  | miR-346 | 0.020676 | 1.064807 |  | |  |  |
|  | miR-937-3p | 0.020828 | 0.749484 |  | |  |  |
|  | miR-5002-3p | 0.021562 | 1.029233 |  | |  |  |
|  | miR-516b-5p | 0.024621 | 1.218233 |  | |  |  |
|  | miR-323a-3p | 0.024647 | 0.575905 |  | |  |  |
|  | miR-376a-3p | 0.025574 | 0.609934 |  | |  |  |
|  | miR-3185 | 0.026174 | 1.96685 |  | |  |  |
|  | miR-136-5p | 0.026724 | 0.578388 |  | |  |  |
|  | miR-6891-3p | 0.027218 | 0.971956 |  | |  |  |
|  | miR-5196-3p | 0.02747 | 1.060088 |  | |  |  |
|  | miR-516b-5p | 0.027624 | 1.200809 |  | |  |  |
|  | miR-6505-3p | 0.030674 | 1.308879 |  | |  |  |
|  | miR-541-3p | 0.03149 | 0.815141 |  | |  |  |
|  |  |  |  | |  | |  |

**Supplementary Table 5: All Higher Expressed miRNA in LSCC Patient Samples via TCGA Analysis**. Differential expression analysis revealed a total number of 64 higher expressed miRNA in Black LSCC patients in TCGA.

|  |  |  |  |  |  |
| --- | --- | --- | --- | --- | --- |
|  | **miRNA** | | **P-Value** | **log(FC)** |  |
|  | miR-3180-5p | | 2.34E-05 | -3.56514 |  |
|  | miR-21-3p | | 0.000794 | -0.66342 |  |
|  | miR-149-5p | | 0.001089 | -1.01261 |  |
|  | miR-141-5p | | 0.001254 | -0.85338 |  |
|  | miR-876-3p | | 0.001402 | -2.80519 |  |
|  | miR-149-3p | | 0.002006 | -1.18755 |  |
|  | miR-30b-3p | | 0.002554 | -0.60359 |  |
|  | miR-200c-5p | | 0.003081 | -0.74309 |  |
|  | miR-3155a | | 0.003242 | -1.53699 |  |
|  | miR-934 | | 0.003395 | -2.36473 |  |
|  | miR-3691-3p | | 0.004493 | -1.52952 |  |
|  | miR-6087 | | 0.005605 | -1.44064 |  |
|  | miR-27a-5p | | 0.006322 | -0.80296 |  |
|  | miR-762 | | 0.006354 | -1.59308 |  |
|  | miR-6742-3p | | 0.006649 | -1.57277 |  |
|  | miR-4524a-3p | | 0.006825 | -1.41181 |  |
|  | miR-4270 | | 0.007384 | -1.34068 |  |
|  | miR-3913-5p | | 0.00808 | -0.56311 |  |
|  | miR-190a-3p | | 0.008571 | -1.42109 |  |
|  | miR-585-3p | | 0.009817 | -1.20301 |  |
|  | miR-335-3p | | 0.009953 | -0.75986 |  |
|  | miR-219b-5p | | 0.010027 | -1.25389 |  |
|  | miR-371b-5p | | 0.010853 | -1.48457 |  |
|  | miR-891a-5p | | 0.0109 | -1.89919 |  |
|  | miR-128-1-5p | | 0.011135 | -0.5085 |  |
|  | miR-5683 | | 0.011569 | -2.25301 |  |
|  | miR-210-5p | | 0.012541 | -0.69138 |  |
|  | miR-9-5p | | 0.012751 | -1.40489 |  |
|  | miR-4289 | | 0.013638 | -2.62336 |  |
|  | miR-7974 | | 0.013759 | -1.0649 |  |
|  | miR-339-5p | | 0.014364 | -0.64934 |  |
|  | miR-5580-3p | | 0.014447 | -1.3084 |  |
|  | miR-6510-5p | | 0.016102 | -1.27505 |  |
|  | miR-511-5p | | 0.016359 | -0.59617 |  |
|  | miR-4488 | | 0.016755 | -1.28437 |  |
|  | miR-9-3p | | 0.017811 | -1.239 |  |
|  | miR-17-3p | | 0.017991 | -0.49733 |  |
|  | miR-27b-5p | | 0.019328 | -0.59447 |  |
|  | miR-6880-5p | | 0.019845 | -1.51731 |  |
|  | miR-6803-5p | | 0.019937 | -1.7097 |  |
|  | miR-23c | | 0.020146 | -0.77194 |  |
|  | miR-1296-3p | | 0.021204 | -1.25172 |  |
|  | miR-4657 | | 0.021309 | -1.26926 |  |
|  | miR-302a-5p | | 0.021692 | -1.15961 |  |
|  | miR-3689a-5p | | 0.021873 | -3.28074 |  |
|  | miR-3180 | | 0.022138 | -2.7983 |  |
|  | miR-3180-3p | | 0.02215 | -2.79831 |  |
|  | miR-592 | | 0.023244 | -1.26265 |  |
|  | miR-196a-3p | | 0.023761 | -1.92034 |  |
|  | miR-6894-3p | | 0.023826 | -1.24223 |  |
|  | miR-4757-5p | | 0.023993 | -1.11806 |  |
|  | miR-3191-3p | | 0.024108 | -1.28982 |  |
|  | miR-449b-5p | | 0.024537 | -2.28725 |  |
|  | miR-6090 | | 0.025241 | -1.55337 |  |
|  | miR-1266-5p | | 0.025578 | -0.76594 |  |
|  | miR-1236-3p | | 0.02571 | -1.09042 |  |
|  | miR-6748-3p | | 0.025731 | -1.16645 |  |
|  | miR-5703 | | 0.025755 | -1.06938 |  |
|  | miR-204-5p | | 0.02588 | -1.61406 |  |
|  | miR-4728-3p | | 0.025906 | -1.07848 |  |
|  | miR-4687-3p | | 0.026332 | -1.33687 |  |
|  | miR-378j | | 0.028006 | -1.66198 |  |
|  | miR-129-1-3p | | 0.02847 | -1.54983 |  |
|  | miR-1180-5p | | 0.028567 | -1.32018 |  |
|  | miR-4327 | | 0.029249 | -1.16163 |  |
|  | miR-4489 | | 0.029717 | -1.07419 |  |
|  | miR-618 | | 0.029947 | -0.80876 |  |
|  | miR-4485-3p | | 0.03034 | -1.02454 |  |
|  |  | |  |  |  |

**Supplementary Table 6: All Lower Expressed miRNA in LSCC Patient Samples via TCGA Analysis**. Differential expression analysis revealed a total number of 68 lower expressed miRNA in Black LSCC patients in TCGA.
