# Supplementary figures and images for: Investigating miR-9 as a mediator in laryngeal cancer health disparities
Source: Front Oncol. 2023 Apr 4;13:1096882. doi: 10.3389/fonc.2023.1096882 (PMC10112398; doi:10.3389/fonc.2023.1096882)

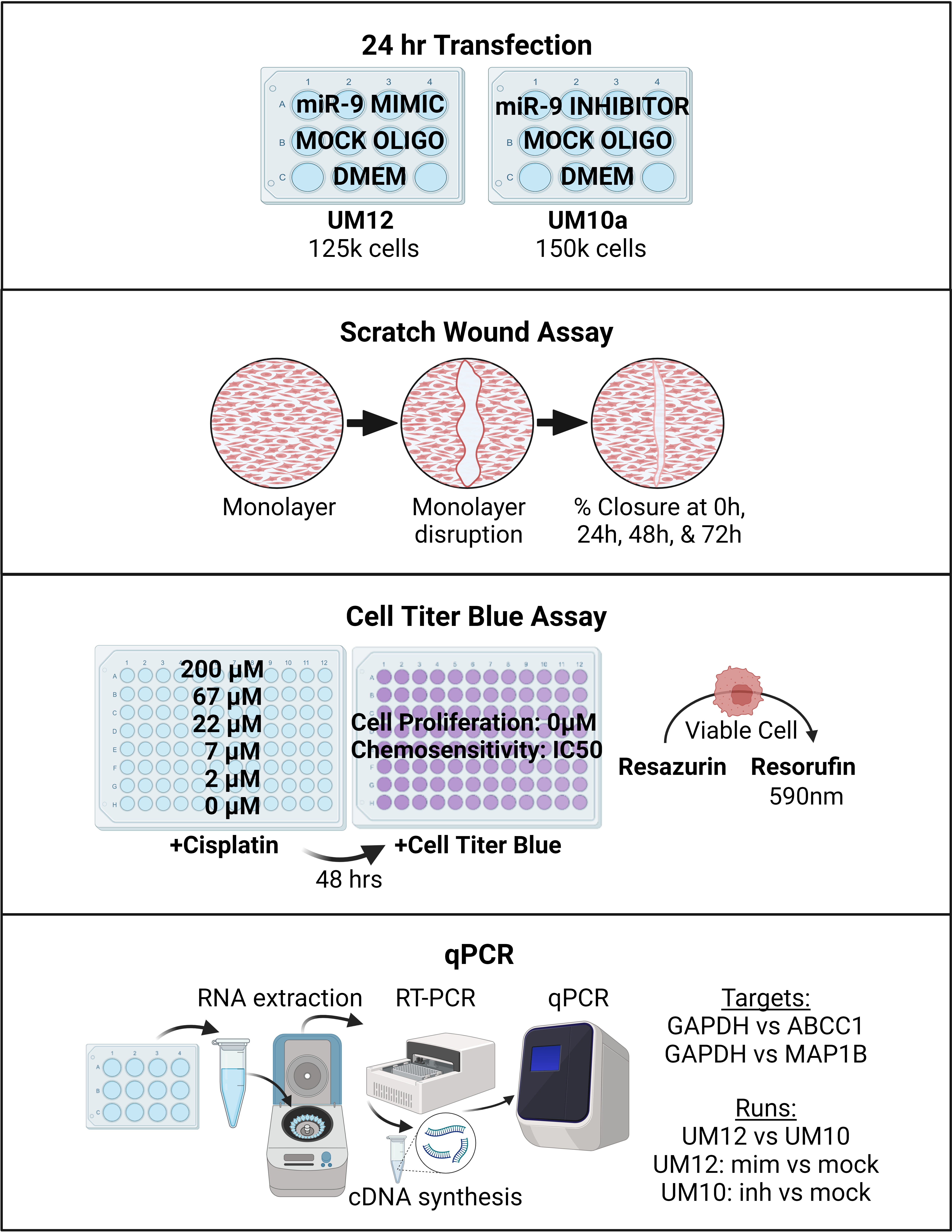

Supplement: Supplementary file 2 [file Image_1.jpeg]
